# Impact of the Recovery on Concentrating Acetic Acid with Low-Pressure Reverse-Osmosis Membranes

**DOI:** 10.3390/membranes11100742

**Published:** 2021-09-28

**Authors:** Giorgio Pratofiorito, Harald Horn, Florencia Saravia

**Affiliations:** 1 Engler-Bunte-Institut, Karlsruhe Institute of Technology (KIT), Water Chemistry and Water Technology, Engler-Bunte-Ring 9, 76131 Karlsruhe, Germany; harald.horn@kit.edu; 2 DVGW Research Center, Water Chemistry and Water Technology, Engler-Bunte-Ring 9a, 76131 Karlsruhe, Germany; saravia@dvgw-ebi.de

**Keywords:** low-pressure reverse osmosis, volatile fatty acids, recovery, osmotic pressure

## Abstract

This work deals with the optimization of the concentration of volatile fatty acids (VFAs) using low-pressure reverse osmosis (LPRO) membranes. Membrane filtration of a synthetic solution simulating the product of biomass hydrolysis was performed. Experiments were run on two flat-sheet XLE membranes under 22 and 25 bar in continuous operation mode. Separation efficiency was evaluated for different recoveries. A correlation between the osmotic pressure of the concentrate and the parameter *R_c_*, representative of the separation efficiency, was found. Under the conditions of the present study and taking into consideration the rejection properties of the applied membrane, a recovery of 33% and 44% is recommendable to maximize the ratio between the concentration of acetate in the concentrate and permeate and thus increase the total reclaim of acetic acid.

## 1. Introduction

The interest in anaerobic digestion processes for biogas production and for the production of platform chemicals (such as lactic and propionic acid) has increased in the last decades [1,2,3]. One process that is being studied in the area of biogas production is the two-stage fermentation process, where biomass is first degraded to simpler organic compounds—mostly volatile fatty acids (VFAs)—via hydrolysis and acidogenesis and later converted into methane by methanogens in a second reactor [4,5]. Electrodialysis, pervaporation, membrane distillation, membrane contactor, forward osmosis, reverse osmosis, and nanofiltration appear to be valid techniques to concentrate these low-molecular-weight substances, increase the organic load, and improve their availability for the last step of the biogas production or for platform chemicals production in general [6,7,8]. Among them, nanofiltration (NF) and low-pressure reverse osmosis (LPRO) have been gaining importance for VFAs recovery in recent years, due to their high retentions with respect to these chemicals. Moreover, they allow the separation of VFAs from other molecules, such as sugars [9]. Table 1 lists different membranes for VFAs recovery applied in the literature.

Some effort has already been made to better understand the mechanisms governing the separation of organic acids from aqueous solutions with NF and LPRO membranes. A previous study showed that 80% of acetic acid can be retained using NF-270 membranes, at a concentration in the feed of 500 mg/L, a pressure of 2.8 bar and pH 7.3 [10]. At pH 7.3, acetic acid is totally dissociated in the solution and can easily be rejected by the negatively charged NF-membranes. However, for solutions with lower pH value, a tighter membrane might be applied in order to achieve higher rejection rates. In fact, pH value plays a double role in the separation of organic acids: it influences the membrane surface charge and the dissociation of the acids. In more acidic solutions, the fraction of dissociated VFAs becomes smaller, and the negative surface charge of the membrane is neutralized by the hydronium in water. Bellona and Drews showed that acetic acid (pK_a_ = 4.76) can be retained up to 60% by the NF-90 membrane at 5.5 bar when the pH is 5.5 [11]. The two researchers also measured the Zeta potential of the membrane, relating a negative Zeta potential with the enhancement of the retention of negative species, confirming the effect of pH value on the rejection of VFAs. Verliefde et al. showed that the acetic acid retention decreased from 98% to 79% when the pH is shifted from 8 to 5 at 25 bar [12]. In this range, the fraction of dissociated acid varies from 100% at pH 8 to 63% at pH 5. Relevant VFAs show similar behavior, as they all have pK_a_ in the range between 4.75 and 4.9.

The presence of inorganic salts can also affect the retention of organic molecules, as pointed out by previous works [13,14,15]. Lactic acid was retained by a Desal-5 DK membrane 30% less when the concentration of NaCl in the feed was increased from 0 to 1 M (pH between 6 and 7) [13]. According to the authors, NaCl screens the electrostatic repulsion between membrane and lactate. This effect becomes more evident for solutes with a molecular weight much smaller than the molecular weight cut-off (MWCO) of the membrane, since for these compounds, charge effect plays a dominant role in the rejection mechanisms. In general, higher ionic strength leads to a neutralization of the membrane, with a consequent reduction of the electrostatic repulsion. Similar results were obtained by Choi et al. [10]. This underlines the importance of considering the matrix when it comes to evaluating the separation performances.

Multicomponent systems can show different retentions than single-component solutions. For instance, acetic acid is retained 11.5% and 7.8% more, when it is mixed with butyric and propionic acid, respectively [16]. Bóna et al. performed a statistical analysis on several runs of filtration of mixtures containing acetic, propionic, and butyric acid using NF and LPRO membranes [17]. As expected, NF showed higher permeability (4.2 L/(m^2^ h bar)) and lower retentions (on average 50%, 64%, and 74% for acetic, propionic, and butyric acid, respectively) compared to LPRO (2.5 L/(m^2^ h bar), 80% retention for acetic, and 84% for propionic and butyric acid) and was more susceptible to changes in the pH.

Another important factor determining the retention of VFAs is the size of the compounds that undergo the filtration. NF/LPRO membranes are dense composite membranes, and the diffusion of smaller chemicals through their active layer is easier than for larger ones. Retention of different molecules in NF/LPRO processes can be linked to several parameters, such as the molecular weight, the equivalent molar diameter, the Stokes diameter, and the calculated molecular diameter [18].

Hydrophobic compounds solvate less and flow more easily through the membrane. [19]. However, in the case of VFAs, hydrophobicity is not determinant. In fact, for short-chain organic acids, the carboxylic polar group is predominant and confers a hydrophilic structure to the compound.

LPRO and NF can concentrate VFAs but are not able to separate them from one another. However, Zhou et al. were able to separate acetic acid from glucose and xylose in a model hydrolysate by using RO98pHt membranes at a pH of about 3 and 20 bar [20,21]. Furthermore, acidic conditions allow acetic acid to flow through the membrane while bigger uncharged molecules such as monosaccharides, for which size exclusion is the dominant effect, are highly retained. They also investigated the effect of temperature on the retention of the three solutes by NF and LPRO membranes, finding that both the monosaccharides and acetic acid are retained less by NF membranes when temperature increases. On the other hand, LPRO membranes showed only a drop in the retention of acetic acid and not of glucose and xylose, resulting in a better separation. A higher temperature implies an increase in the diffusion of the solutes and a change in the thin-layer structure, where pore size becomes larger.

In spiral wound modules, the choice of the recovery has consequences on the overall efficiency of the separation. The use of continuous pilot plants permits one to simulate the behavior of a spiral wound module when operated at a desired recovery. Moreover, the effect of recovery on the efficiency of the concentration of VFAs has not been deeply investigated so far. In previous studies on continuous pilot plants, experiments were only conducted at a fixed recovery [22,23].

The aim of this study is to evaluate the effect of the recovery on the LPRO of a model solution defined to simulate a hydrolysate obtained from biomass hydrolysis. An attempt was made to explain the mechanisms involved, in order to better understand the optimal condition to run the process. The scope was to find out whether a high or limited recovery is desirable, preventing in this way the loss of the acetic acid in the permeate.

## 2. Materials and Methods

### 2.1. Experimental Setup

**Figure 1:**
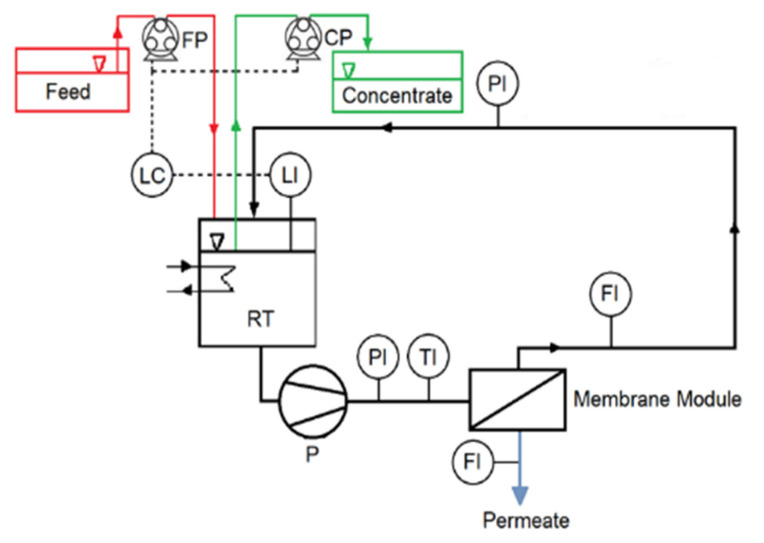
Schematic representation of the system. FP: Feed pump; CP: concentrate pump; RT: recirculation tank; P: pressure; T: temperature; F: flow; and L: level.

The experiments were performed in a continuously operated laboratory setup (PS Prozesstechnik GmbH, Basel, Switzerland). It consisted of two cross-flow flat sheet membrane modules (active surface 0.279 × 0.1 m^2^, channel thickness 1 mm) connected in parallel (Figure 1). A photograph of one module is provided in the Supplementary Material (Figure S1) as well as one of the setup (Figure S2). A similar module was used by West et al. [24]. Two equal pieces of XLE (FilmTec Dupont, Wilmington, DE, USA), a polyamide thin-film composite membrane, were used in this work. According to the manufacturer, this membrane has a stabilized salt rejection of 99% (500 ppm NaCl in the feed stream) with a flux of 7.4 L/(m^2^ h bar). The desired concentrate has to be fed to a methane reactor and must contain a high amount of acetate and, at the same time, not extremely high salinity. Therefore, a tighter membrane was not chosen. Permeate flux, temperature, and pressure were measured continuously throughout the experiments. Experiments were run at 22 ± 0.5 and at 25 ± 0.5 bar. These two pressures were chosen on the basis of the expected osmotic pressure of the feed (10 bar) and the optimal pressure needed to achieve an adequate membrane flux. The temperature was controlled by a heat exchanger placed in the recirculation tank (RT in Figure 1) and kept at 37 ± 1 °C, the temperature in hydrolysis reactors operated at mesophilic conditions.

The cross-flow velocity in the feed channel was set at 0.2 m/s. The system can be considered as a black box. The feed is the only entering stream, while the concentrate and permeate are the two streams leaving the system. However, there was an internal recirculation, which made it possible to test different recoveries. Anytime the volume inside the recirculation tank (RT) decreased below 4.8 L a level control unit turned on two pumps, FP and CP. FP pumped fresh medium into RT, while CP pumped the mixture from RT at a lower flowrate than FP. The level control unit turned them off when the level was reached again. In this way, the volume in RT remained practically constant, since the quantity of liquid delivered by FP equaled that pumped by CP plus the volume leaving the system as permeate. The ratio between the flowrates of the feed (*Q_f_*) and the solution withdrawn from RT (*Q_c_*) was varied, in order to evaluate the influence of the recovery on the separation efficiency of the whole process. This ratio was referred to as the concentration factor (CF, Equation (1)). Equation (2) clarifies the relation between recovery and concentration factor. The higher CF, the higher the recovery.

(1) $$\mathrm{CF}=\frac{Q_{f}}{Q_{c}}$$

(2) $$Recovery\;\left(\%\right)=\left(1-\frac{1}{\mathrm{CF}}\right)\cdot 100$$

### 2.2. Feed Solution

The feed solution was prepared with 13 g/L acetic acid, 5.5 g/L calcium acetate, 4.8 g/L NaOH, 3.5 g/L KOH, and 0.6 g/L NH_4_Cl. These values correspond to a total acetate concentration of 17 g/L. The pH was 5.4 ± 0.1, and the electrical conductivity was 17 ± 0.5 mS/cm. It was decided to use only acetic acid because previous works showed that its retention is equal or smaller than that of other relevant VFAs such as propionate, butyrate, and lactate [17,25]. At this pH, acetate accounts for 80% of total acetic acid, according to the Henderson–Hasselbalch equation. The formulation of the feed was performed based on the hydrolysates, described in the literature by Kumanowska et al. and Ravi et al. [4,5].

### 2.3. Sampling and Analytics

The compositions of the feed, the concentrate, and the permeate were determined on a daily basis. Dissolved organic carbon (DOC) and total nitrogen (TN) were measured with a carbon analyzer (TOC-L CPH, Shimadzu Corporation, Kyōto, Japan). Since the only nitrogen source in the solution was NH_4_Cl, the TN value was proportional to the ammonium concentration. Inductively coupled plasma optical emission spectroscopy (ICP-OES) (Agilent, Model 5110, Santa Clara, CA, USA) was used to determine the concentrations of cations (Ca^2+^, Na^+^, K^+^). The concentration of Cl^−^ was measured with ion chromatography (Metrohm, 790 Personal IC, Herisau, Switzerland).

### 2.4. Development of Parameters

The focus of the present work is the maximization of acetate in the concentrate of a membrane process (LPRO) for its further use. To compare the quantity of acetate being withdrawn from RT as concentrate with the one in the permeate, the ratio between the concentration of acetate in the two streams (*R_c_*) was defined as follows:

(3) $$R_{c}=\frac{C_{c}}{C_{p}}\;$$

where *C_c_* (mg/L) is the concentration in the concentrate and *C_p_* (mg/L) the one in the permeate. The higher *R_c_*, the higher the efficiency of the separation.

Another important parameter is the ratio between the acetate flowrate in the concentrate and in the permeate:

(4) $$R_{w}=\frac{W_{c}}{W_{p}}\;$$

where *W_c_* (mg/min) is the mass flowrate in the concentrate and *W_p_* (mg/min) is the one in the permeate. *R_w_* simply represents the relation between the overall amounts of acetate flowing in the two streams and indicates how much acetate is being “lost” in the permeate.

The osmotic pressure π was calculated based on the concentrations of ions measured with the different techniques according to:

(5) $$\pi =RT\sum_{k=1}^{n}i_{k}C_{k}\;$$

where *T* is the temperature (K), *C_k_* the concentration of the component *k* (mol/L), *n* the number of components, *i_k_* the Van’t Hoff coefficient of the component *k*, and *R* is the universal gas constant ((L bar)/(mol K)). The osmotic pressure was calculated assuming that the concentrations at the membrane surface mimicked the ones in the bulk, neglecting concentration polarization. The approach used for the calculation of the osmotic pressure is a linear approach, i.e., the Van’t Hoff coefficients are independent of the concentration and equal the charges of the considered ions. For acetate, the coefficient equals 0.8, since it is partially dissociated, as discussed in Section 2.2. The assumption of linearity in the range of concentration of the present work is validated by Nagy et al. [26]. In the case of NaCl, as soon as the concentration reaches 4 M, this assumption does not hold anymore.

The acetic acid mass balance over the whole system is represented by Equation (6):

(6) $$Q_{A}^{F}=Q_{A}^{P}+Q_{A}^{C}\;$$

where $Q_{A}^{F}$ is the flowrate of acetic acid entering the system by FP, $Q_{A}^{P}$ the flowrate of acetic acid leaving the system in the permeate, and $Q_{A}^{C}$ the one being withdrawn by CP. The term concerning the permeate sampling does not appear in the balance, since permeate was continuously disposed and its collection did not interfere with the operation of the plant.

## 3. Results and Discussion

### 3.1. Experiment at 22 Bar

**Figure 2:**
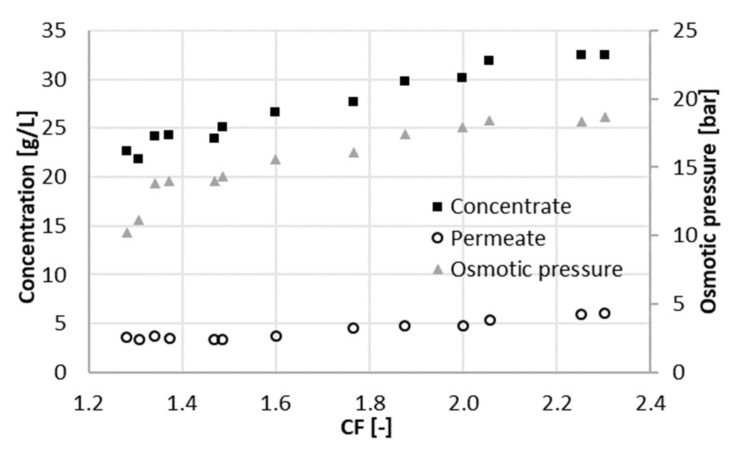
Osmotic pressure and acetate concentrations in the concentrate and in the permeate at different concentration factors CF (22 bar).

**Figure 3:**
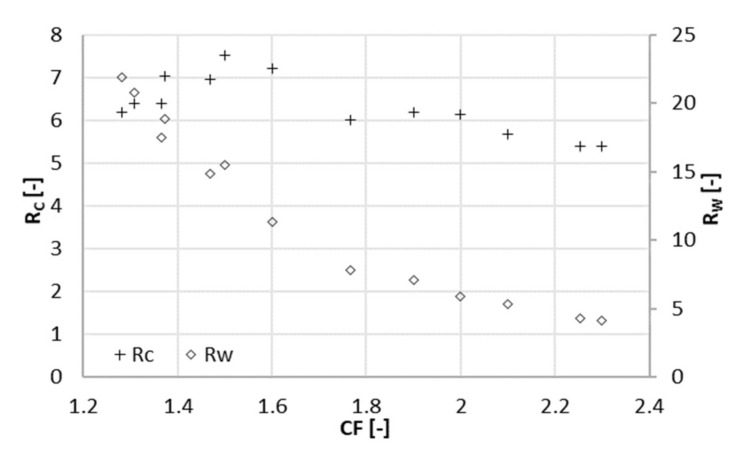
Concentration ratio (*R_c_*) and mass ratio (*R_w_*) at different concentration factors CF (22 bar).

**Figure 4:**
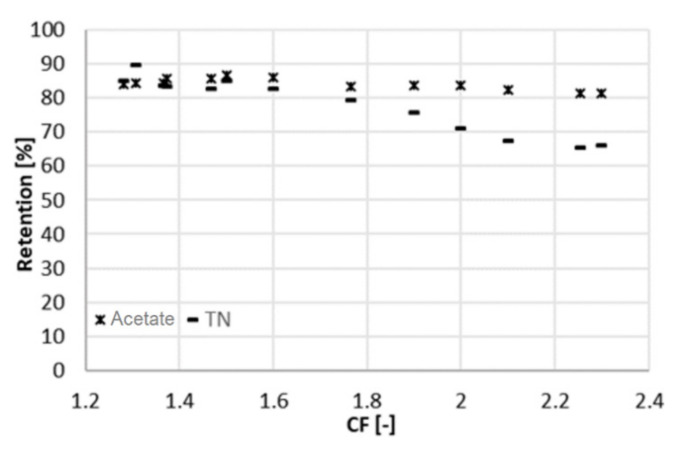
Retention of acetic acid and ammonium (measured as TN) at different CF (22 bar).

Data were collected over a period of 15 days during the filtration experiment at 22 bar. After setting CF to the desired value, the sampling was performed at least one day later, to give the system time to stabilize. The average volumetric flowrate of the feed varied significantly, namely from 10 mL/min (at a CF of 2.3) to 62 mL/min (at a CF of 1.3). This was caused by the strong dependence of the permeate flux on CF. Increasing CF implied a lower flux through the membrane due to the high concentration in the feed channel. In turn, the level in RT decreased more slowly, and the feed and concentrate were pumped in and out less frequently. The total flowrate of the permeate withdrawn from the system varied from 5.8 to 14.5 mL/min. The average volumetric flowrate of the concentrate varied accordingly from 4.3 to 47 mL/min. Figure 2, Figure 3 and Figure 4 depict the concentration of acetic acid in the concentrate and in the permeate, as well as the osmotic pressure, the parameters *R_c_* and *R_w_*, and the retention of ammonium and acetic acid as a function of the concentration factor.

The concentration of acetic acid in the permeate increases with CF. This was a consequence of the higher concentration in the feed, which led to a higher osmotic pressure (Figure 2). A higher concentration in the feed resulted in a higher flux of solute through the membrane. A higher osmotic pressure indicated at the same time a decline of the effective pressure, i.e., the driving force for water flux through the membrane. Thus, the concentration of acetic acid in the permeate turned out to be greater, and the permeability decreased with the concentration factor, since the effective applied pressure decreased.

A positive contribution to the parameter *R_c_* (Figure 3) was given by the increase in the acetate concentration in the concentrate when the concentration factor ranged from 1.3 to 1.5 (Figure 2). When the concentration factor was greater than 1.5, *R_c_* showed a decrease. The concentration of acetate in the concentrate at a CF of 2.3 was 30% higher than at a CF of 1.5. In contrast, concentration in the permeate almost doubled in the same range. In other words, the increment in the concentration of acetate (and ions in general) in the concentrate did not compensate anymore for the one in the permeate. This was reflected in the parameter *R_c_*, which had its maximum value at a concentration factor of 1.5, corresponding to a recovery of 33%.

*R_w_*, the ratio between the flowrates of acetate in the concentrate and the permeate, decreased in the whole range of CF tested values. Nevertheless, the decrease slowed down for a concentration factor greater than 2.

The results concerning the retention are consistent with the literature. XLE was already used under similar conditions and at comparable acetate concentrations, retaining 85% of acetate [15]. The retention (Figure 4) is negatively affected by an increase in the concentration factor. This was due to the higher concentration of the solution facing the membrane, with the consequences described above. However, the decline in retention was more pronounced for the total nitrogen (as an indicator of ammonium concentration) than for acetate. This might be due to the positive charge of ammonium, which is more easily pushed through the negatively charged membrane, as the ionic strength in the feed channel increased.

According to previous experiments, fouling/biofouling may occur only after 16 days of operation, under the same conditions of the present study. Consequently, fouling is assumed to play no role for the experiments presented in this work.

Concentration polarization could be expected to play a role in determining the permeate flux and consequently the parameters *R_c_* and *R_w_*. Nevertheless, under the present conditions, no clear influence is expected. The Reynolds number in the channel (570) was much higher than the one associated to a concentration polarization factor (CPF) of 1.1 by Salcedo-Díaz et al. [27]. Additionally, Jung et al. showed that at a cross-flow velocity of 0.2 m/s the CPF can even assume lower values [28].

### 3.2. Experiment at 25 Bar

**Figure 5:**
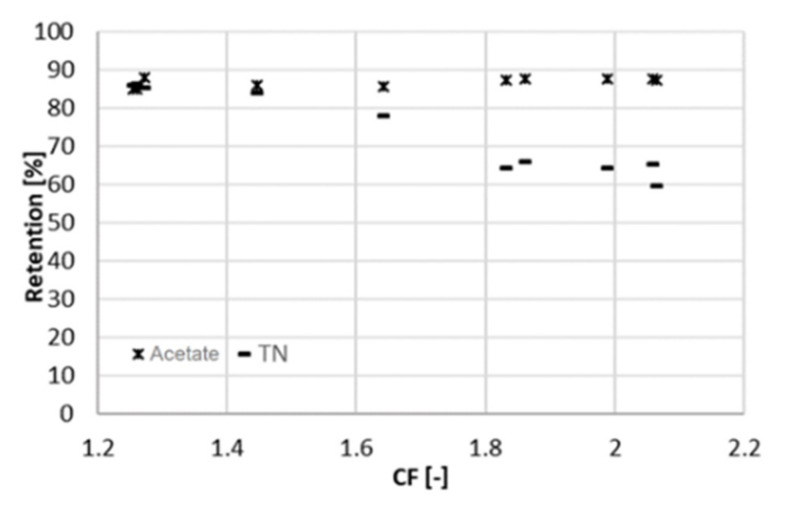
Retention of acetic acid and ammonium (measured as TN) at different concentration factors CF (25 bar).

A similar experiment to the one presented above was run at 25 bar to evaluate the influence of pressure changes on rejection and membrane performance. The system behaved similar as at 22 bar. This time, the overall duration of the experiment was 10 days, with a sampling interval of one day. The average volumetric flowrate of the feed varied from 9 mL/min (at a CF of 2.1) to 55 mL/min (at a CF of 1.3). In the same range, the sum of the two permeate flowrates varied from 4.7 to 11 mL/min and the flowrate of the concentrate from 4.3 to 44 mL/min. At higher pressure, the retention of acetate is slightly higher and remains practically constant (88%) throughout the range of CF (Figure 5). A lower retention (75%) was obtained by Bóna et al. with LPRO membrane at the same pH, although at lower pressure of 6 bar [17]. Choi et al. also observed lower acetic retentions at pH 5.5; however, looser NF membranes were used in their study [10].

**Figure 6:**
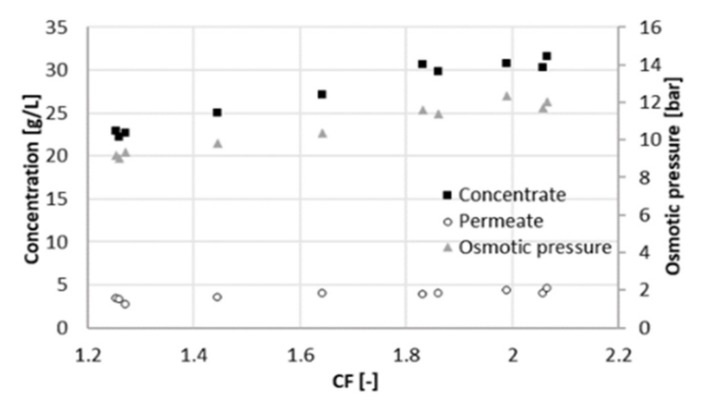
Osmotic pressure and acetate concentrations in the concentrate and in the permeate at different concentration factors CF (25 bar).

**Figure 7:**
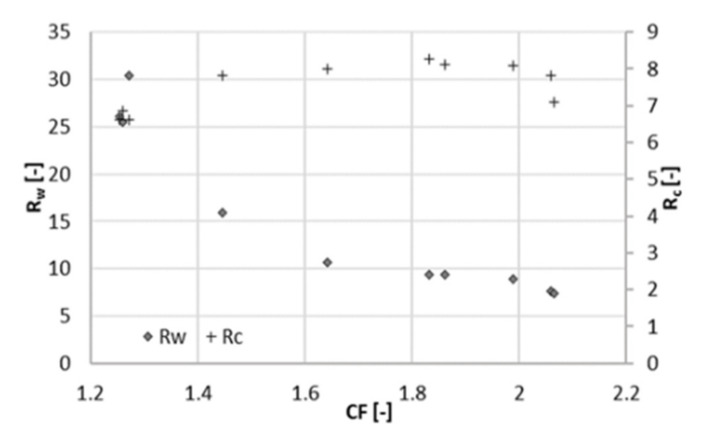
Concentration ratio (*R_c_*) and mass ratio (*R_w_*) at different concentration factors CF (25 bar).

Retention of total nitrogen decreases dramatically (from 85% to 60%), similarly as at 22 bar. Likewise, this can be explained by the Donnan effect. The increase in the ionic strength in the feed channel negatively affects the retention of chloride. Consequently, ammonium must flow to the permeate side to maintain charge equilibrium. At the maximum recovery observed, the concentration of acetic acid in the permeate was 4 g/L at 25 bar and 6 g/L at 22 bar (Figure 6), resulting in a larger *R_c_*. The concentration of acetate in the concentrate was comparable between both experiments, around 32 g/L. Due to the better rejection, the maximum of *R_c_* shifts to the right and reached a higher value (Figure 7). For values of CF higher than 1.8 (recovery of 44%), *R_c_* started decreasing again, and the concentration of acetate in the concentrate did not increase proportionally to the one in the permeate (Figure 5). Hence, a higher applied pressure meant a higher optimal recovery. It must be therefore pointed out that the optimal recovery does not only depend on the characteristics of the solution but also on the operation conditions and the rejection properties of different membranes that change the boundaries of the process.

### 3.3. Economic Considerations

In order to qualitatively assess whether an increase of 3 bar of the operating pressure may have a beneficial effect on the overall economics of the process, *R_c_* must be considered together with the recovery. At different pressures, the maximum *R_c_* (*R_c_^max^*) was found at different recoveries. This means that the pressure giving the highest *R_c_^max^* is not necessarily the best option. For 22 bar, the optimal recovery was 33% (*R_c_^max^* = 7.5), and for 25 bar, it was 44% (*R_c_^max^* = 8.3). That is, the separation efficiency was higher in the second case, but at a higher recovery, more permeate is produced. The parameter *R_w_* can better clarify this aspect. *R_w_* is about 15 and at 22 bar and 10 at 25 bar, meaning that at 25 bar 50% more acetate is lost in the permeate than at 22 bar. In other words, at 25 bar, a better separation efficiency can be achieved, but this fact is compensated by the high amount of produced permeate, causing a considerable loss of solute.

Besides, the difference between the two pressures (3 bar) is more than 10% of the total applied pressure. Thus, it is conceivable to expect that the energy consumption differs by the same order of magnitude. In conclusion, with an operational cost roughly one tenth higher and a loss of solute in the permeate 50% greater, the operation at 25 bar appears to be less advantageous.

## 4. Conclusions

Acetic acid was concentrated using a low-pressure reverse-osmosis membrane. The separation efficiency was assessed at different recoveries by varying the concentration factor CF. The results showed that above a certain value of recovery (33% at 22 bar and 44% at 25 bar), the efficiency of the separation (represented by *R_c_*) started to decrease. As the recovery increased, the solution in the feed channel was more concentrated. As a consequence of the higher concentration gradient along the membrane, a considerable amount of solute was lost in the permeate, and the total reclamation of acetic acid decreased. Therefore, it can be concluded that recovery should only be increased up to a certain extent, even despite other effects such as fouling and concentration polarization.

Concerning the operating pressure, a preliminary economic consideration showed that the higher costs needed to provide a higher pressure are not justified by a better overall performance. A higher pressure can indeed result in a better separation efficiency. On the other hand, with a higher permeate flux, a greater amount of solute can be lost.

## Figures and Tables

**Table 1:** Membranes used for VFAs recovery.

Membrane	Material	VFA Recovered	NF/LPRO	Manufacturer	Reference
NF-90	Polyamide	Acetic acid	NF	FilmTec-Dow	[11]
NF-270	Polyamide	Formic, acetic, butyric acids	NF	FilmTec-Dow	[10]
ES10	Aromatic polyamide	Formic, acetic, butyric acids	NF	Nitto Denko	[10]
Desal-5 DK	Cross-linked aromatic polyamide	Lactic acid	LPRO	GE	[13]
SW30	Polyamide	Acetic, propionic, butyric acids	LPRO	FilmTec-Dow	[17]
dNF40	Polyethersulfone	Acetic, propionic, butyric acids	NF	NX Filtration	[17]
RO98pHt	Aromatic polyamide	Acetic acid	LPRO	Alfa Laval	[20,21]
Trisep TS80	Aromatic polyamide	Formic, acetic, lactic acids	NF	Trisep	[12]
Desal HL	Cross-linked aromatic polyamide	Formic, acetic, lactic acids	NF	GE	[12]
XLE	Polyamide	Acetic, propionic, butyric acids	LPRO	FilmTec-Dow	[15]

## Data Availability

The data that support the findings of this study are available on request from the corresponding author.

## Supplementary Materials

The following are available online at https://www.mdpi.com/article/10.3390/membranes11100742/s1, Figure S1: Flat sheet module. Feed side on the right and permeate side on the left, Figure S2: LPRO setup. The two modules are placed vertically (on the right).
