# Supplementary material for: Impact of the Recovery on Concentrating Acetic Acid with Low-Pressure Reverse-Osmosis Membranes
Source: Membranes (Basel). 2021 Sep 28;11(10):742. doi: 10.3390/membranes11100742 (PMC8540121; doi:10.3390/membranes11100742)
Supplement: Supplementary file 1 [file membranes-11-00742-s001.zip › membranes-1374715.supplementary.pdf]

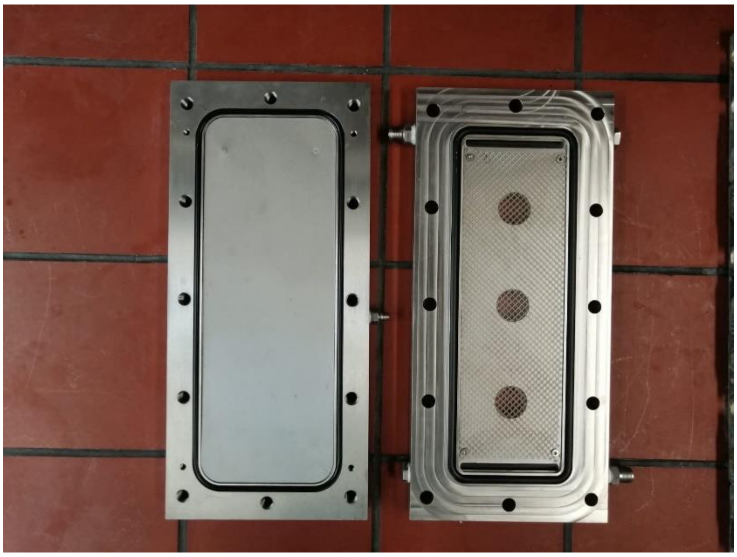

Figure S1. Flat sheet module. Feed side on the right and permeate side on the left.

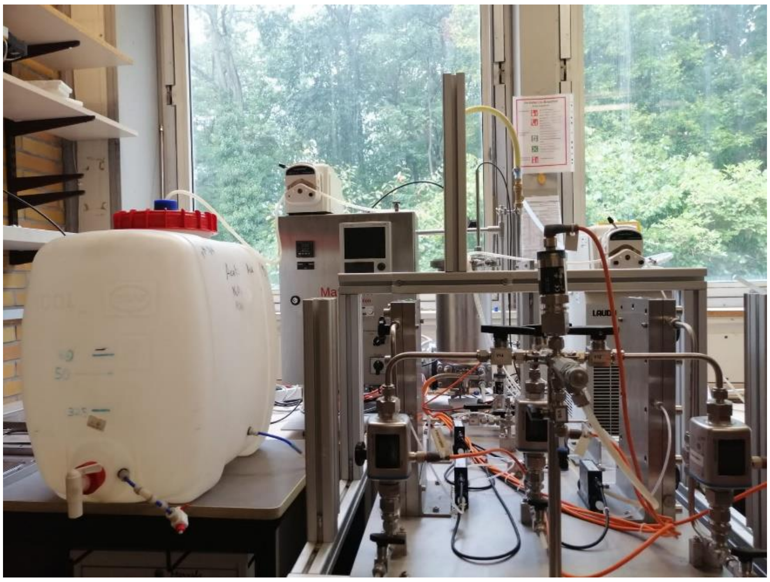

Figure S2. LPRO setup. The two modules are placed vertically (on the right).
